# Dietary changes during the Great Recession in Portugal: comparing the 2005/2006 and the 2014 health surveys

**DOI:** 10.1017/S136898001800410X

**Published:** 2019-03-22

**Authors:** Ricardo Alves, Julian Perelman

**Affiliations:** Escola Nacional de Saúde Pública, Centro de Investigação em Saúde Pública, Universidade NOVA de Lisboa, Av. Padre Cruz, Lisboa 1600–560, Portugal

**Keywords:** Dietary habits, Recession, Mediterranean diet

## Abstract

**Objective:**

We aimed at analysing changes in consumption of selected food groups in the Portuguese population before and after the Great Recession, which hit the country between 2008 and 2013.

**Design:**

We used pooled cross-sectional data from the Portuguese National Health Interview Surveys of 2005/2006 and 2014. We modelled the probability of consumption of soup, fish, meat, potatoes/rice/pasta, bread, legumes, fruit, vegetables and sweets/desserts, as a function of the year, controlling for age, sex and education, using logistic regressions. Then, we stratified the analysis by age group and education level. Analyses were adjusted for survey weights.

**Setting:**

Portugal (2005/2006 to 2014).

**Participants:**

Adults (*n* 43273) aged 25–79 years.

**Results:**

From 2005/2006 to 2014, there was a significantly lower consumption of fish, soup, fruit and vegetables. Conversely, the consumption of legumes and sweets/desserts was significantly higher in 2014. The changes in the selected food groups were consistent across most education levels. Among people aged 65 years or above, there were no significant changes in most foods, except an increase in the consumption of legumes and sweets/desserts. In contrast, people aged 25–39 and 40–64 years significantly decreased their intakes of fish and soup and increased their consumption of sweets/desserts.

**Conclusions:**

The consistent results across education levels suggest that changes in dietary habits are not linked to the economic downturn. By contrast, our findings suggest a shift away from foods commonly linked to the Mediterranean diet, particularly among younger people.

Studies analysing the impact of economic recessions have shown a positive effect of weakened economic condition on some health risk behaviours. Unemployment and the loss of income were observed, for example, to contribute to the reduction in alcohol and tobacco consumption^(^ ^1^ ^–^ ^4^ ^)^. However, the effect on eating behaviours is controversial and research on this topic has produced mixed results. Some evidence suggests that shorter working hours are associated with the reduction of fast foods and prepared processed foods^(^ ^5^ ^)^. During the recent recession in the USA, evidence suggested that consumption of foods prepared away from home declined and overall diet quality improved slightly^(^ ^6^ ^)^. Another recent study observed different results, stating that the high state-level unemployment had no significant impact on patterns of home food preparation and away-from-home eating, concluding that adults from low-income populations were resistant to food-related behaviour change, even during major economic recessions^(^ ^7^ ^)^.

According to the Portuguese National Institute of Statistics, the recent economic recession led to a steep increase in unemployment in Portugal, which almost doubled between 2005 and 2014^(^ ^8^ ^)^. This contributed to the rise in rates of material deprivation† among the overall population, and particularly among working-age individuals from 18 to 64 years old^(^ ^8^ ^)^. In 2014, the prevalence of individuals with some type of food insecurity reached nearly 46% of the whole population. Of this group, about 16% were considered as having either a moderate or severe level of food insecurity^(^ ^9^ ^)^. These data are especially relevant considering that Portugal has one of the highest ratios of income inequality in Europe^(^ ^10^ ^)^. Hence, the recent economic recession in Portugal might have influenced the eating behaviours of some sub-populations disproportionately.

Diet patterns and access to adequate nutrition are influenced by complex structural and socio-economic factors^(^ ^11^ ^)^. For instance, in low-income populations, time scarcity is a major obstacle^(^ ^7^ ^,^ ^12^ ^)^. Balancing work, child care or daily commute is time-consuming and people struggle to have time to prepare healthy meals at home^(^ ^13^ ^,^ ^14^ ^)^, resorting to ready-to-eat processed foods that are generally less healthy^(^ ^15^ ^,^ ^16^ ^)^. In the USA and Britain, there is evidence suggesting a discrepancy in prices between nutrient-rich foods and less nutritious alternatives, where less healthy foods tend to be more resistant to inflation^(^ ^16^ ^–^ ^18^ ^)^. This disparity in prices may present an important constraint to a healthier diet among low-income groups.

On the one hand, we may suspect the impoverishment of the population to have improved the dietary pattern, due to shorter working hours providing more time for home-made foods. On the other hand, we may hypothesise a decline in the consumption of healthy foods, which are generally more expensive and thus less affordable in a period of economic downturn. Finally, it may be that these phenomena occurred simultaneously in different sub-populations; for example, the decreased consumption of healthy foods due to price might have been more likely among the worse-off, while the greater availability to cook might have been more likely among the younger people.

Meanwhile, there have been significant changes in diet habits in Europe, particularly in Portugal^(^ ^19^ ^)^. Research suggests that in the last decades Portugal is drifting away from the Mediterranean diet, which is associated with better health outcomes. This diet is characterised by high intakes of vegetables, fruits and whole grains, moderate consumption of fish and white meat, and low consumption of red meat, processed meats and foods rich in sugars^(^ ^20^ ^–^ ^23^ ^)^. Studies from 2007 and 2009 indicate that there has been a decrease in intakes of vegetables, fish and wholegrain products, and an increased intake of processed foods^(^ ^19^ ^,^ ^24^ ^)^.

In the present study, we examined the changes in consumption of selected food groups in Portugal, comparing the 2005/2006 and 2014 health surveys and analysing their socio-economic patterning. This aim is particularly significant considering the role of poor diet as a major risk factor for non-communicable diseases, such as CVD, diabetes and cancer^(^ ^25^ ^–^ ^28^ ^)^. In recent years, increasing evidence from observational studies and randomised trials has shown an association between an unbalanced diet and intermediate outcomes including high prevalence of hypertension^(^ ^29^ ^)^, obesity^(^ ^30^ ^)^ and ischaemic stroke risk^(^ ^31^ ^)^.

## Methods

### Study design and population

We did a secondary analysis of cross-sectional data from the National Health Interview Surveys of 2005/2006 and 2014. These surveys are cross-sectional studies based on representative samples of the non-institutionalised population living in Portugal^(^ ^32^ ^,^ ^33^ ^)^. The data from National Health Interview Surveys are collected by the National Institute for Statistics and are available on demand for research purposes. The sample for the 2005/2006 survey was selected from the mother sample of the Population and Housing Census data from 2001. The methodology consisted of a systematic selection of primary units (areas) based on probability proportional to the number of households^(^ ^33^ ^)^. The 2014 survey followed a regional and multistage stratified sampling scheme, in which the primary units (areas) were systematically selected based on probability proportional to the number of households, and the secondary units (households) were based on random sampling within primary units^(^ ^34^ ^)^.

The National Health Interview Survey of 2005/2006 was conducted from 7 February 2005 to 7 February 2006, with face-to-face interviews of all individuals residing in each of the housing units included. A total of 15 457 families were interviewed, corresponding to 41 193 residents with a response rate of 76% for the national territory^(^ ^33^ ^)^. The responses from the Portuguese National Health Interview Survey 2014 were collected between September and December 2014 through face-to-face interviews and via the Internet. A total of 18 204 valid responses were obtained, corresponding to an overall response rate of 80%^(^ ^32^ ^)^. Note that the individuals interviewed in the 2005/2006 and 2014 questionnaires are not the same; that is, the analysis was based on repeated cross-sections and not on cohorts. Given that the methodology used to design the samples was similar, we opted to pool the data from the two surveys.

We only considered people above 25 and below 79 years of age. Younger people were not included to avoid considering people who did not conclude their education; and older people because the survey did not consider institutionalised persons, so that the sample might be biased for this sub-population.

### Outcomes

All dependent variables were dichotomised: consumption of meat (‘yes’ and ‘no’); consumption of soup (‘yes’ and ‘no’); consumption of fish (‘yes’ and ‘no’); consumption of potatoes/rice/pasta (‘yes’ and ‘no’); consumption of bread (‘yes’ and ‘no’); consumption of legumes (‘yes’ and ‘no’); consumption of sweets/desserts (‘yes’ and ‘no’); consumption of fruit (‘yes’ and ‘no’ for the 2005/2006 survey only); and consumption of vegetables (‘yes’ and ‘no’ for the 2005/2006 survey only). These classes are the result of questions made in both 2005/2006 and 2014 surveys: participants were asked if they had eaten any of these foods groups in the main meals of the previous day. However, in the 2014 survey, fruit and vegetable intakes were measured in a different way; that is, people were asked about the number of days they consumed these foods in the last week (‘7 days a week’; ‘6–4 days a week’; ‘3–1 days a week’; ‘less than 1 a week’; ‘never’), instead of whether they had eaten fruits and vegetables or not, like in 2005. To identify fruit and vegetable consumption, we considered the percentage of individuals reporting to have consumed fruit and vegetables in the 24h recall, for the 2005/2006 sample; and the number of individuals reporting to have consumed fruits and vegetables on 7d/week, for the 2014 sample. We acknowledge that the different measures in 2005/2006 and 2014 potentially bias the comparison, but the alternative was to remove fruit and vegetables from the analyses, which would represent a serious limitation given the relevance of these nutrients. Hence, the results for these two variables should be viewed with caution.

The consumption of soup could be seen as a possible proxy for the intake of vegetables. There is also evidence linking soup intake to a decreased risk of obesity in both genders in Portugal^(^ ^35^ ^)^. The main negative aspect with soup consumption in Portugal is the high average sodium levels^(^ ^36^ ^)^.

The food groups were selected due to their overall nutritional value, protective and detrimental health effects, and relevance to the Mediterranean diet^(^ ^20^ ^,^ ^21^ ^,^ ^37^ ^)^. The use of these food groups can be presented as significant public health indicators for the impact of dietary habits on the Portuguese population.

In this context, evidence shows that different dietary patterns or specific foods can lead to a considerable variation in the risk of premature death^(^ ^38^ ^,^ ^39^ ^)^. For example, a recent meta-analysis suggested that while the increasing intake of red and processed meats or sugar-sweetened beverages had a positive association with a higher risk of all-cause mortality, the consumption of whole grains, fruits, vegetables, legumes or fish had an inverse association^(^ ^39^ ^)^.

### Explanatory variables and covariates

The explanatory variable was the year of the interview (2005/2006 and 2014), coded as a dichotomous variable. Covariates included sex (female; male), educational level (no education (0–3 years of education); pre-primary education (4 years of education); primary education (9 years of education); secondary education (12 years of education); tertiary education (>12 years of education)) and age (25–39 years; 40–64 years; ≥65 years).

### Statistical analysis

To analyse the primary outcome, we used logistic regression to model the consumption of soup, fish, meat, potatoes/rice/pasta, bread, legumes, fruit, vegetables and sweets/desserts, as a function of the year, controlling for age, sex and education. We adjusted OR and their 95% CI for the independent variables, establishing the level of significance at *P*<0·05^(^ ^40^ ^)^.

We then stratified the analysis with the same variables by education level and age, controlling for age/education level and sex. We divided the education level into three different groups: no education/pre-primary education; primary education/secondary education; and tertiary education. We also stratified our population by three distinct age groups: 25–39, 40–64 and ≥65 years. The different associations of dietary intake and year were tested through interacting the year variable with education levels and with age categories. All analyses were adjusted for survey weights.

Analyses were conducted using the statistical software package Stata version 13.

## Results

A total of 43 273 observations were included in the analysis. Almost two-thirds of all participants were part of the 2005/2006 survey (Table 1). The percentage of individuals with tertiary education increased from 10·0% in 2005/2006 to 17·4% in 2014.

**Table 1:** Baseline characteristics of participants from the Portuguese National Health Interview Surveys of 2005/2006 and 2014

	2005/2006		2014		Total	
Variable	*n*	%	*n*	%	*n*	%
Sex						
Female	14 908	53·0	8475	56·0	23 383	54·0
Male	13 236	47·0	6654	44·0	19 890	46·0
Age (years)						
25–39	7353	26·1	3354	22·2	10 707	24·7
40–64	13 886	49·3	7692	50·8	21 578	49·9
65–79	6905	24·5	4083	27·0	10 988	25·4
Survey year	28 144	65·0	15 129	35·0	43 273	100·0
Educational level						
No education/pre-primary education	19 399	69·0	7911	53·3	27 310	52·3
Primary education/secondary education	5918	21·0	4584	30·3	10 502	24·3
Tertiary education	2815	10·0	2634	17·4	2634	12·6

**Fig.1:**
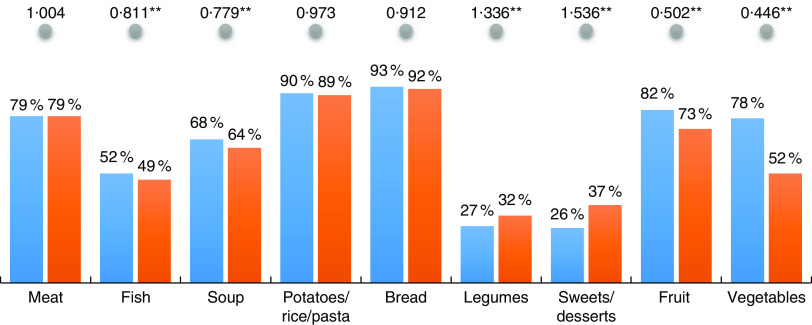
(colour online) Unadjusted percentage consumption of key foods in 2014 (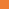) *v*. 2005/2006 (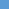), and adjusted OR (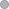) for 2014 *v*. 2005/2006, controlling for age/education level and sex using binary logistic regression, among Portuguese adults (*n* 43 273) aged 25–79 years participating in the National Health Interview Surveys of 2005/2006 and 2014. *P<0·05, **P < 0·01

In 2014, there was an overall decrease in the consumption of fish, soup, fruit and vegetables and an increase in the consumption of legumes and sweets/desserts (Fig. 1). Compared with 2005/2006, there was in 2014 a significantly lower consumption of fish (49 *v*. 52%, OR=0·81, *P*<0·01), soup (64 *v*. 68%, OR=0·78, *P*<0·01), fruit (73 *v.* 82%, OR=0·50, *P*<0·01) and vegetables (52 *v*. 78%, OR=0·45, *P*<0·01). Conversely, the consumption of legumes (32 *v*. 27%, OR=1·34, *P*<0·01) and sweets/desserts (37 *v.* 26%, OR=1·54, *P*<0·01) were significantly higher in 2014. There were no significant changes in the consumption of meat, bread and potatoes/rice/pasta between the 2005/2006 and 2014 surveys.

**Fig.2:**
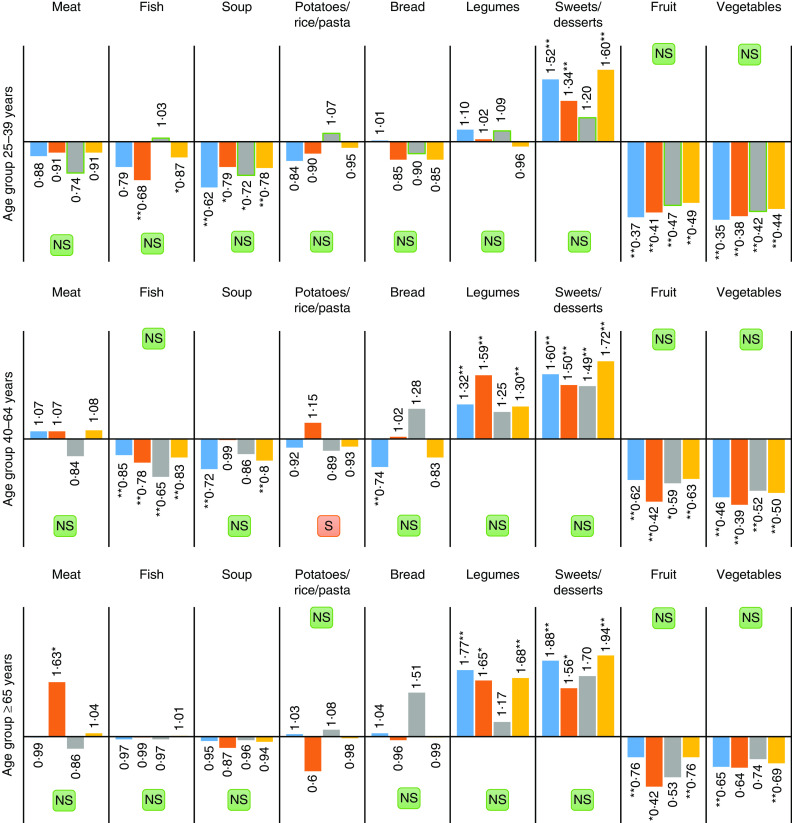
(colour online) Adjusted OR for consumption of key foods in 2014 *v*. 2005/2006 among different age groups, stratified by education level (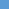, no education/pre-primary education; 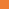 primary education/secondary education; 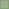 tertiary education; 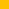 all groups), controlling for sex using binary logistic regression, among Portuguese adults (*n* 43 273) aged 25–79 years participating in the National Health Interview Surveys of 2005/2006 and 2014. *P<0·05, **P < 0·01 (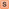, significant differences in changes across sub-populations, *P*<0·01; 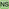 non-significant differences in changes across sub-populations, *P*>0·01)

Between 2005/2006 and 2014, the variation patterns in the consumption of key foods were similar among younger generations (age groups 25–39 years and 40–64 years). Within these groups, there was a significantly lower consumption of fish, soup, fruit and vegetables and a higher intake of sweets/desserts (Fig. 2). The only difference between these groups was in the consumption of legumes, which did not change among the youngest people (25–39 years). For people aged ≥65 years, there was a significant increase in the intake of legumes (OR=1·68, *P*<0·01) and sweets/desserts (OR=1·94, *P*<0·01). These changes were mostly consistent across education groups (we did not observe significant differences across educational categories), except for the consumption of soup, which decreased more among the lowest educated people aged 40–64 years.

When comparing sub-populations with different education levels, we observed an increase in the consumption of sweets among the three categories, and of legumes among people with low and middle education. The consumption of soup declined only among the low educated, and of fish among those with middle education. Changes were mostly consistent across age groups, except for the increase in legumes, which was higher among the low- and middle-educated elderly, and the decrease in soup, which was greater among the low-educated younger people.

## Discussion

### Key findings

During a time frame of approximately 9 years (2005/2006 to 2014), there were significant changes in the intake of key food groups in Portugal: there was a generalised decrease in the consumption of soup, fish, fruit and vegetables, and an overall increase in consumption of legumes and sugar-containing foods. Meat, potatoes/rice/pasta and bread did not experience significant changes from 2005/2006 to 2014.

This pattern was observed among those aged 25–64 years, except the increase in legumes intake, which was not found among those aged 25–39 years. Among the elderly aged ≥65 years, the decrease in fish and soup consumption was not observed. These findings were consistent across education categories, except soup that decreased more among the low-educated people aged 40–64 years.

### Interpretation

The changes in consumption of selected food groups had similar patterns across education groups, except the consumption of soup, which did not change significantly during this period among the lowest educated people aged 40–64 years. Whereas the drop in fish intake could be attributed to financial constraints, this is not the case for soup, which is generally cheaper and whose consumption also declined. Furthermore, the increase in sweets may be in part explained by their affordability. However, the similar results observed in most education levels calls this explanation into question.

The more dramatic reduction in the overall consumption of fruit and vegetables from 2005/2006 to 2014 may derive from the different outcome measures used in the 2005/2006 and 2014 surveys. Thus, we will refrain from drawing strong conclusions about this decline. However, it is worth noticing that the variation in fruit and vegetable intake follows a similar pattern across education groups as found for the remaining foods included in the present study.

People aged ≥65 years did not significantly reduce the intake of any food, except fruit and vegetables. This is despite the elderly being a particularly vulnerable group, characterised by a high percentage of low-educated people with lower average earnings^(^ ^41^ ^,^ ^42^ ^)^, and thus more exposed to an economic recession.

In Portugal, there is a robust association between income and level of education. The most educated people tend to have higher average earnings compared with the lowest educated^(^ ^41^ ^,^ ^43^ ^)^. Therefore, the findings could suggest that most of these changes in the consumption of selected food groups were widespread, independently from one’s level of education and income. However, we cannot support the premise that loss of income was a driving factor behind the observed habits changes if we consider that the recent economic recession disproportionately affected the Portuguese population, where families with the lowest income lost almost twice as much of their earnings compared with families with higher revenues^(^ ^44^ ^)^.

A study conducted in Italy that examined adherence to a Mediterranean diet during the recent recession observed different results^(^ ^45^ ^,^ ^46^ ^)^. Wealth and education were major determinants of the changes in dietary patterns. Less affluent groups (mainly living in urban areas) and the elderly had a higher decrease in adherence to this diet, therefore suggesting a role of the economic downturn on eating behaviours.

Although the present study did not specifically measure adherence to the Mediterranean diet, our results may also be interpreted as resulting from the long-term decline in this adherence, rather than from short-term effects of the economic downturn. For example, the reduction in intakes of fish and soup and the increase in sweets are likely related to a reduction in adherence to the Mediterranean diet from 2005/2006 to 2014. This trend is in line with previous studies that showed Portugal has been drifting away from the Mediterranean diet in the last decades^(^ ^19^ ^,^ ^24^ ^)^. The only exception was in the consumption of legumes, which increased significantly during this period.

The reduction of fish and soup was felt more prominently among younger people, which seems to indicate that these younger generations were less resistant to food-related behaviour changes. The exception to this trend was the consumption of legumes, which did not increase among the younger individuals. Again, this finding may be interpreted as a more pronounced reduction in adherence to the Mediterranean diet within younger generations when compared with the elderly.

This trend towards poorer adherence to this diet among younger people in Southern European countries is well documented in a recent overview of studies^(^ ^47^ ^)^. Most of the research analysed in that review showed a tendency for lower intakes of vegetables, fruits and fish and increased consumption of products with low nutrient density like soft drinks and sweets.

One should also consider the role of the food supply and availability of certain types of food product^(^ ^48^ ^,^ ^49^ ^)^. In Mediterranean countries, some research from the beginning of the 21st century has pointed out the increasing energy availability from non-Mediterranean foods like animal fats and sugar products, contrasting with the fall in energy availability provided from plant proteins and cereals^(^ ^48^ ^)^. Technical changes in the food industry, rapid urbanisation and increasing dissemination of ready-to-eat outlets should also be considered and studied as possible drivers of the observed dietary changes^(^ ^49^ ^,^ ^50^ ^)^. Furthermore, additional studies are needed to understand if these changes could be influenced by economic constraints or other barriers such as lack of time, knowledge or skills to prepare healthier meals, particularly among younger generations.

### Limitations

The overall results of the present study should be viewed with caution. For instance, we could not evaluate the general quality of the products consumed and if there were changes to less/more healthy options of the same proteins/vegetables/carbohydrates over time. The collected data from the Portuguese National Health Interview Surveys of 2005/2006 and 2014 did not include questions regarding the types of foods consumed. The surveys did not specify whether people were eating meals prepared at home or resorting to away-from-home foods or processed foods. Hence, it was not possible to evaluate the general quality of the foods consumed.

This is also true regarding each food individually, particularly for the consumption of meat and fish: we did not know the overall quality of these products, mainly if those were processed meat and fish, which is crucial to a more comprehensive assessment of the Mediterranean diet. This missing information is key to future analysis of diet patterns during economic recessions. We may hypothesise that economic constraints could lead vulnerable groups to less healthy options, switching from fresh products to cheaper high-energy-dense alternatives.

Another limitation concerns the methods used to assess food intake. In the Portuguese National Health Interview Surveys, participants were asked what they ate in the previous day, relying on the participant’s memory to measure the consumption of the selected food groups. Dietary records or food diaries are more comprehensive and detailed dietary assessment methods that report a high validity and precision^(^ ^51^ ^,^ ^52^ ^)^. Nevertheless, these methods are less feasible in large representative samples of a national population. Working on a representative sample of the Portuguese population is a strength that counterbalances this limitation.

The present study’s results for the consumption fruit and vegetable variables should be carefully considered and analysed. As stated in the ‘Methods’ section, these variables were measured differently between the 2004/2005 and the 2014 surveys. It is likely that this may cause a bias towards a reported lower consumption in the 2014 sample.

Since it was not possible to access data about the income of study participants, conclusions regarding average earnings were inferred from the level of education, which are closely related in Portugal, but not equivalent^(^ ^41^ ^,^ ^43^ ^)^.

Additionally, there is also evidence suggesting that methods using self-report responses to measure dietary intake could be biased by social desirability or social approval^(^ ^53^ ^,^ ^54^ ^)^.

Finally, stratified analyses may provide non-significant differences between socio-economic groups due to small samples; in some cases, we observed differences between groups in the magnitude of estimates, which were however not significant. We opted to not emphasise these differences in magnitude, in a conservative approach, but larger databases may provide different findings.

### Policy implications

These observed changes in eating habits in Portugal support the urge for a review of past/current public health policies related to dietary habits. Public policies that tackle the structural factors behind the reduction in adherence to the Mediterranean diet should be put in place to reverse the trends shown in the present study.

## Conclusions

Our study shows that there has been an overall reduction in the consumption of fish, soup, fruit and vegetables, and an increase in meat and sweets. Although the Mediterranean diet was not directly assessed in the current study, the observed pattern may be indicative of a decline in adherence to this diet in the Portuguese population from 2005/2006 to 2014, particularly among younger people. The decline was felt consistently across educational levels, which differs from the available evidence from Southern Europe. Given the close link between education and income in Portugal and the disproportionate impact of economic crisis among the worse-off, these results seem to indicate that the observed changes are not linked to the economic downturn. Therefore, our findings may be interpreted as resulting from long-term declining adherence to the Mediterranean diet.

However, these results should be viewed with caution and future studies should take into consideration a more detailed account of the types of foods consumed. Bearing in mind the evidence on changing diet habits in Western societies, the role of the food industry, food supply and urbanisation should be studied as possible drivers for these changes.

## Footnotes

† The percentage of persons living in households where there was a lack of at least three of the nine items of economic deprivation (including not financially able to have a meat or fish meal or a vegetarian equivalent).

## References

[1] Kawachi I (2012) Economic crisis and smoking behaviour: prospective cohort study in Iceland. Bus Manag J 2, 1–7. 10.1136/bmjopen-2012-001386 PMC3488705 23048059

[2] Ruhm CJ (2003) Good times make you sick. J Health Econ 22, 637–658. 12842319 10.1016/S0167-6296(03)00041-9

[3] Ruhm CJ (2005) Healthy living in hard times. J Health Econ 24, 341–363. 15721049 10.1016/j.jhealeco.2004.09.007

[4] Freeman DG (1999) A note on economic conditions and alcohol problems. J Health Econ 18, 659–668. 10.1016/s0167-6296(99)00005-3 10621370

[5] Mercan MA (2013) A research note on the relationship between long working hours and weight gain for older workers in the United States. Res Aging 36, 557–567. 25651510 10.1177/0164027513510324

[6] Todd JE (2014) *Changes in Eating Patterns and Diet Quality among Working-Age Adults, 2005–2010. Economic Research Report* no. 161. Washington, DC: US Department of Agriculture, Economic Research Service.

[7] Smith LP, Ng SW & Popkin BM (2014) Resistant to the recession: low-income adults’ maintenance of cooking and away-from-home eating behaviors during times of economic turbulence. Am J Public Health 104, 840–846. 24625145 10.2105/AJPH.2013.301677 PMC3987573

[8] Insituto Nacional de Estatística (2015) *Anuário Estatístico de Portugal 2014: enquadramento populacional*. Lisboa: Instituto Nacional de Estatística.

[9] Direção-Geral de Saúde (2015) Programa Nacional para a Promoção da Alimentação Saudável. Lisboa: Direção-Geral de Saúde.

[10] European Commission (2017) *Key Figures on Europe* – 2017 Edition. Luxembourg: Publications Office of the European Union.

[11] Azétsop J & Joy TR (2013) Access to nutritious food, socioeconomic individualism and public health ethics in the USA: a common good approach. Philos Ethics Humanit Med 8, 16. 24165577 10.1186/1747-5341-8-16 PMC4231366

[12] Davis GC & You W (2011) Not enough money or not enough time to satisfy the Thrifty Food Plan? A cost difference approach for estimating a money-time threshold. Food Policy 36, 101–117.

[13] Rose D (2007) Food Stamps, the Thrifty Food Plan, and meal preparation: the importance of the time dimension for US nutrition policy. J Nutr Educ Behav 39, 226–232. 17606249 10.1016/j.jneb.2007.04.180

[14] Beatty TK, Nanney MS & Tuttle C (2014) Time to eat? The relationship between food security and food-related time use. Public Health Nutr 17, 66–72. 23340255 10.1017/S1368980012005599 PMC10282377

[15] Monteiro CA, Levy RB, Claro RM et al. (2011) Increasing consumption of ultra-processed foods and likely impact on human health: evidence from Brazil. Public Health Nutr 14, 5–13. 21211100 10.1017/S1368980010003241

[16] Drewnowski A (2004) Obesity and the food environment: dietary energy density and diet costs. Am J Prev Med 27, 3 Suppl., 154–162. 15450626 10.1016/j.amepre.2004.06.011

[17] Monsivais P & Drewnowski A (2007) The rising cost of low-energy-density foods. J Am Diet Assoc 107, 2071–2076. 18060892 10.1016/j.jada.2007.09.009

[18] Monsivais P, Mclain J & Drewnowski A (2010) The rising disparity in the price of healthful foods: 2004–2008. Food Policy 35, 514–520. 25411518 10.1016/j.foodpol.2010.06.004 PMC4234177

[19] da Silva R, Bach-Faig A, Raidó Quintana B et al. (2009) Worldwide variation of adherence to the Mediterranean diet, in 1961–1965 and 2000–2003. Public Health Nutr 12, 1676–1684. 19689839 10.1017/S1368980009990541

[20] Sofi F, Cesari F, Abbate R et al. (2008) Adherence to Mediterranean diet and health status: meta-analysis. BMJ 337, a1344. 18786971 10.1136/bmj.a1344 PMC2533524

[21] Sofi F (2009) The Mediterranean diet revisited: evidence of its effectiveness grows. Curr Opin Cardiol 24, 442–446. 19550306 10.1097/HCO.0b013e32832f056e

[22] Fidanza F, Alberti A, Lanti M et al. (2004) Mediterranean Adequacy Index: correlation with 25-year mortality from coronary heart disease in the Seven Countries Study. Nutr Metab Cardiovasc Dis 14, 254–258. 15673059 10.1016/s0939-4753(04)80052-8

[23] Castro-Quezada I, Román-Viñas B & Serra-Majem L (2014) The Mediterranean diet and nutritional adequacy: a review. Nutrients 6, 231–248. 24394536 10.3390/nu6010231 PMC3916858

[24] Rodrigues SSP, Caraher M, Trichopoulou A et al. (2007) Portuguese households’ diet quality (adherence to Mediterranean food pattern and compliance with WHO population dietary goals): trends, regional disparities and socioeconomic determinants. Eur J Clin Nutr 62, 1263–1272. 17671445 10.1038/sj.ejcn.1602852

[25] World Cancer Research Fund & American Institute for Cancer Research (2007) Food, Nutrition, Physical Activity, and the Prevention of Cancer: A Global Perspective. Washington, DC: AICR.

[26] Schwingshackl L, Hoffmann G, Lampousi A-M et al. (2017) Food groups and risk of type 2 diabetes mellitus: a systematic review and meta-analysis of prospective studies. Eur J Epidemiol 32, 363–375. 28397016 10.1007/s10654-017-0246-y PMC5506108

[27] Hunter DJ & Reddy KS (2013) Noncommunicable diseases. N Engl J Med 369, 1336–1343. 24088093 10.1056/NEJMra1109345

[28] Ezzati M & Riboli E (2013) Behavioral and dietary risk factors for noncommunicable diseases. N Engl J Med 369, 954–964. 24004122 10.1056/NEJMra1203528

[29] Karppanen H & Mervaala E (2006) Sodium intake and hypertension. Prog Cardiovasc Dis 49, 59–75. 17046432 10.1016/j.pcad.2006.07.001

[30] Malik VS, Schulze MB & Hu FB (2006) Intake of sugar-sweetened beverages and weight gain: a systematic review. Am J Clin Nutr 84, 274–288. 16895873 10.1093/ajcn/84.1.274 PMC3210834

[31] Boden-Albala B, Elkind MS V, White H et al. (2009) Dietary total fat intake and ischemic stroke risk: the Northern Manhattan study. Neuroepidemiology 32, 296–301. 19246935 10.1159/000204914 PMC2824586

[32] Instituto Nacional de Estatística & Instituto Nacional de Saúde Doutor Ricardo Jorge (2015) *Inquérito Nacional de Saúde 2014: mais de metade da população com 18 ou mais anos tinha excesso de peso*. Lisboa: Instituto Nacional de Estatística e Instituto Nacional de Saúde Doutor Ricardo Jorge.

[33] Instituto Nacional de Estatística & Instituto Nacional de Saúde Doutor Ricardo Jorge (2009) *Inquérito Nacional de Saúde 2005/2006*. Lisboa: Instituto Nacional de Estatística e Instituto Nacional de Saúde Doutor Ricardo Jorge.

[34] Instituto Nacional de Estatística (2016) *Inquerito Nacional de Saúde 2014*. Lisboa: Instituto Nacional de Estatística.

[35] Moreira P & Padra P (2006) Educational, economic and dietary determinants of obesity in Portuguese adults: a cross-sectional study. Eat Behav 7, 220–228. 16843224 10.1016/j.eatbeh.2005.08.008

[36] Gonçalves C (2006) Occupational safety and hygiene sodium content in vegetable soups prepared outside the home: identifying the problem. In *Proceedings of the International Symposium on Occupational Safety and Hygiene*, pp. 278–281. Guimarães, Portugal: Portuguese Society of Occupational Safety and Hygiene.

[37] Portugal, Ministério da Saúde, Direcção-Geral da Saúde (2005) *Princípios para uma Alimentação Saudável*. Lisboa: Direcção Geral de Saúde.

[38] Schwingshackl L & Hoffmann G (2015) Diet quality as assessed by the Healthy Eating Index, the Alternate Healthy Eating Index, the Dietary Approaches to Stop Hypertension score, and health outcomes: a systematic review and meta-analysis of cohort studies. J Acad Nutr Diet 115, 780–800. 25680825 10.1016/j.jand.2014.12.009

[39] Schwingshackl L, Schwedhelm C, Hoffmann G et al. (2017) Food groups and risk of all-cause mortality: a systematic review and meta-analysis of prospective studies. Am J Clin Nutr 105, 1462–1473. 28446499 10.3945/ajcn.117.153148

[40] Marôco J (2014) *Estatística com o SPSS Statistics*, 6a ed. Pêro Pinheiro, Portugal: Report Number.

[41] PORDATA (2017) *Rendimento Médio Equivalente: por nível de escolaridade (Euro)*. Lisboa: Fundação Francisco Manuel dos Santos.

[42] Instituto Nacional de Estatística (2017) *Rendimento e Condições de Vida 2016: 2,6 milhões de residentes em risco de pobreza ou exclusão social em 2016*. Lisboa: Instituto Nacional de Estatística.

[43] Cantante F (2014) Desigualdades economicas multi-escalares: Portugal no contexto global. Anal Soc 49, 534–566.

[44] Rodrigues CF, Figueiras R & Junqueira V (2016) *Desigualdade do Rendimento e Pobreza em Portugal: as consequências sociais do programa de ajustamento.* Lisboa: Fundação Francisco Manuel dos Santos.

[45] Bonaccio M, Di Castelnuovo A, Bonanni A et al. (2014) Decline of the Mediterranean diet at a time of economic crisis. Results from the Moli-sani study. Nutr Metab Cardiovasc Dis 24, 853–860. 24819818 10.1016/j.numecd.2014.02.014

[46] Bonaccio M, Bes-Rastrollo M, de Gaetano G et al. (2016) Challenges to the Mediterranean diet at a time of economic crisis. Nutr Metab Cardiovasc Dis 26, 1057–1063. 27524802 10.1016/j.numecd.2016.07.005

[47] Grosso G & Galvano F (2016) Mediterranean diet adherence in children and adolescents in southern European countries. NFS J 3, 13–19.

[48] Balanza R, García-Lorda P, Pérez-Rodrigo C et al. (2007) Trends in food availability determined by the Food and Agriculture Organization’s food balance sheets in Mediterranean Europe in comparison with other European areas. Public Health Nutr 10, 168–176. 17261226 10.1017/S1368980007246592

[49] Alexandratos N (2006) The Mediterranean diet in a world context. Public Health Nutr 9, 111–117. 16512957 10.1079/phn2005932

[50] Drewnowski A & Popkin BM (1997) The nutrition transition: new trends in the global diet. Nutr Rev 55, 31–43. 9155216 10.1111/j.1753-4887.1997.tb01593.x

[51] Freisling H, Ocké MC, Casagrande C et al. (2015) Comparison of two food record-based dietary assessment methods for a pan-European food consumption survey among infants, toddlers, and children using data quality indicators. Eur J Nutr 54, 437–445. 24916012 10.1007/s00394-014-0727-7

[52] Ortega RM, Perez-Rodrigo C & Lopez-Sobaler AM (2015) Métodos de evaluación de la ingesta actual: registro o diario dietético. Nutr Hosp 31, 38–45. 25719769 10.3305/nh.2015.31.sup3.8749

[53] Schoeller DA (1995) Limitations in the assessment of dietary energy intake by self-report. Metabolism 44, 18–22. 10.1016/0026-0495(95)90204-x 7869932

[54] Hebert JR, Clemon L, Pbert L et al. (1995) Social desirability bias in dietary self-report may compromise the validity of dietary intake measures. Int J Epidemiol 24, 389–398. 7635601 10.1093/ije/24.2.389

